# Assessment of Frailty Index at 66 Years of Age and Association With Age-Related Diseases, Disability, and Death Over 10 Years in Korea

**DOI:** 10.1001/jamanetworkopen.2022.48995

**Published:** 2023-03-02

**Authors:** Jieun Jang, Heewon Jung, Jaeyong Shin, Dae Hyun Kim

**Affiliations:** 1 Hinda and Arthur Marcus Institute for Aging Research, Hebrew SeniorLife, Harvard Medical School, Boston, Massachusetts; 2 Department of Hospital Administration, Yonsei University Graduate School of Public Health, Seoul, Korea; 3 Division of Geriatrics, Department of Internal Medicine, Asan Medical Center, University of Ulsan College of Medicine, Seoul, Korea; 4 Department of Preventive Medicine, Yonsei University College of Medicine, Seoul, Korea; 5 Department of Policy Analysis and Management, College of Human Ecology, Cornell University, Ithaca, New York

## Abstract

**Importance:**

A frailty index has been proposed as a measure of aging among older individuals. However, few studies have examined whether a frailty index measured at the same chronologic age at younger ages could forecast the development of new age-related conditions.

**Objective:**

To examine the association of the frailty index at 66 years of age with incident age-related diseases, disability, and death over 10 years

**Design, Setting, and Participants:**

This retrospective nationwide cohort study used the Korean National Health Insurance database to identify 968 885 Korean individuals who attended the National Screening Program for Transitional Ages at 66 years of age between January 1, 2007, and December 31, 2017. Data were analyzed from October 1, 2020, to January 2022.

**Exposures:**

Frailty was defined using a 39-item frailty index ranging from 0 to 1.00 as robust (<0.15), prefrail (0.15-0.24), mildly frail (0.25-0.34), and moderately to severely frail (≥0.35).

**Main Outcomes and Measures:**

The primary outcome was all-cause death. Secondary outcomes were 8 age-related chronic diseases (congestive heart failure, coronary artery disease, stroke, type 2 diabetes, cancer, dementia, fall, and fracture) and disability qualifying for long-term care services. Cox proportional hazards regression and cause-specific and subdistribution hazards regression were used to examine hazard ratios (HRs) and 95% CIs for the outcomes until the earliest of date of death, the occurrence of relevant age-related conditions, 10 years from the screening examination, or December 31, 2019.

**Results:**

Among the 968 885 participants included in the analysis (517 052 women [53.4%]), the majority were classified as robust (65.2%) or prefrail (28.2%); only a small fraction were classified as mildly frail (5.7%) or moderately to severely frail (1.0%). The mean frailty index was 0.13 (SD, 0.07), and 64 415 (6.6%) were frail. Compared with the robust group, those in the moderately to severely frail group were more likely to be women (47.8% vs 61.7%), receiving medical aid insurance for low income (2.1% vs 18.9%), and less active (median, 657 [IQR, 219-1133] vs 319 [IQR, 0-693] metabolic equivalent task [min/wk]). After adjusting for sociodemographic and lifestyle characteristics, moderate to severe frailty was associated with increased rates of death (HR, 4.43 [95% CI, 4.24-4.64]) and new diagnosis of all chronic diseases, including congestive heart failure (adjusted cause-specific HR, 2.90 [95% CI, 2.67-3.15]), coronary artery disease (adjusted cause-specific HR, 1.98 [95% CI, 1.85-2.12]), stroke (adjusted cause-specific HR, 2.22 [95% CI, 2.10-2.34]), diabetes (adjusted cause-specific HR, 2.34 [95% CI, 2.21-2.47]), cancer (adjusted cause-specific HR, 1.10 [95% CI, 1.03-1.18]), dementia (adjusted cause-specific HR, 3.59 [95% CI, 3.42-3.77]), fall (adjusted cause-specific HR, 2.76 [95% CI, 2.29-3.32]), fracture (adjusted cause-specific HR, 1.54 [95% CI, 1.48-1.62]), and disability (adjusted cause-specific HR, 10.85 [95% CI, 10.00-11.70]). Frailty was associated with increased 10-year incidence of all the outcomes, except for cancer (moderate to severe frailty adjusted subdistribution HR, 0.99 [95% CI, 0.92-1.06]). Frailty at 66 years of age was associated with greater acquisition of age-related conditions (mean [SD] conditions per year for the robust group, 0.14 [0.32]; for the moderately to severely frail group, 0.45 [0.87]) in the next 10 years.

**Conclusions and Relevance:**

The findings of this cohort study suggest that a frailty index measured at 66 years of age was associated with accelerated acquisition of age-related conditions, disability, and death over the next 10 years. Measuring frailty at this age may offer opportunities to prevent age-related health decline.

## Key Points

**Question:**

Is a frailty index measure at 66 years of age associated with incident age-related chronic disease, disability, and death?

**Findings:**

This nationwide cohort study of 968 885 Korean individuals found that a frailty index measured at 66 years of age was associated with greater rates of death, new major age-related diseases, and disability-qualifying long-term care services over 10 years. Frailty was associated with faster acquisition of new age-related conditions.

**Meaning:**

These findings suggest that measuring frailty at 66 years of age provides an aging trajectory that one is likely to follow, which may be useful to mitigate health decline associated with aging.

## Teaser

This cohort study examines the association of a frailty index at 66 years of age with development of a range of major age-related diseases, disability, and death over 10 years in the population of Korea.

## Introduction

Aging is characterized by deterioration of physiologic function and a decline in intrinsic capacity to tolerate stressors, thereby increasing vulnerability to disease, disability, and death. The World Health Organization calls for integrated care for older people on the basis of intrinsic capacity.^1^ Measuring intrinsic capacity from a clinical assessment may give opportunities to identify people at risk for accelerated aging and test interventions to slow aging.^2,3^ A deficit-accumulation frailty index, which quantifies the burden of age-related, clinically detectable health deficits,^4^ has been proposed as a measure of vulnerability and aging. Previous research^5,6,7,8,9,10^ has shown that a frailty index could estimate death, disability, and health care cost, as well as poor outcomes after acute illnesses and stressful treatments, in older populations with a wide age spectrum. However, it remains uncertain whether a frailty index measured at the same chronologic age at 66 years of age, when most people are free of major illnesses and disability, can identify those at risk for development of a range of age-related chronic diseases and disability. Answering this question may lend support to the frailty index as an integrative measure of aging. Additionally, it will help early identification of people who may benefit from clinical and public health interventions to promote healthy aging.^11,12,13,14,15^

We conducted a retrospective nationwide cohort study of the Korean population to examine the association of the frailty index at 66 years of age with development of a range of major age-related diseases, disability, and death over 10 years. All Korean citizens are invited to a standardized clinical assessment as part of the National Screening Program for Transitional Ages at 66 years of age.^16^ We leveraged the screening clinical database and the Korean National Health Insurance claims database to test the hypothesis that a higher frailty index at 66 years of age would be associated with a greater incidence of age-related conditions and death.

## Methods

### Data Sources

This cohort study was approved by the Institutional Review Board at Ajou University Health System, Suwon, Korea. The study followed the Strengthening the Reporting of Observational Studies in Epidemiology (STROBE) reporting guideline. Korean National Health Insurance Sharing Service system approved use of the National Health Information Database and made anonymized data available to the researchers. Therefore, informed consent from the participants was waived. Through the Korean National Health Insurance Corporation research program, we accessed the National Screening Program for Transitional Ages database (2007-2017) linked to the Korean National Health Insurance database (2004-2019) for a 35% random sample of adults who turned 66 years of age between January 1, 2007, and December 31, 2017 (n = 1 612 991). The National Screening Program for Transitional Ages is a nationwide screening program that was established in 2007 for early detection of chronic diseases for all Korean citizens aged 40 to 66 years.^16^ The screening examination takes place at government-certified clinics, hospitals, and public health centers. The examination at 66 years of age includes questionnaires on lifestyle, medical history, and functional status; physical examination, including a 3-m timed up-and-go test and assessment of vision and hearing; screening tests for depression and dementia; and biometric and laboratory measurements. The Korean National Health Insurance database contains information on sociodemographic characteristics, medical service claims, diagnosis codes from the *International Statistical Classification of Diseases and Related Health Problems, Tenth Revision* (*ICD-10*), health care utilization, and long-term care insurance claims.^17^

### Study Population

Of 1 612 991 enrollees of the Korean National Health Insurance who turned 66 years of age between 2007 and 2017, we included those who had compete sociodemographic information and participated in the screening program (n = 1 005 884). We excluded those who had duplicate records (n = 3336) and insufficient data (<80% of the necessary items) for the calculation of the frailty index (n = 33 663). Our final cohort included 968 885 enrollees whose frailty index could be calculated (eFigure 1 in Supplement 1). This cohort included both community-dwelling older adults and long-term care residents. Compared with nonparticipants, participants were more likely to have self-employed insurance (66.7% vs 57.9%), living outside the capital area (61.7% vs 57.1%), and free of disability (99.5% vs 97.8%) (eTable 1 in Supplement 1). Compared with participants with insufficient data for frailty index calculation, those with sufficient data were more likely to be insured by medical aid (3.4% vs 1.6%), living outside the capital area (61.7% vs 52.9%), and never smokers (68.5% vs 61.1%) (eTable 1 in Supplement 1).

### Measurement of Frailty and Other Characteristics

We followed the standard procedure by Searle et al^18^ (2008) to build our frailty index. Variables were included in calculation of the frailty index if they were associated with health status; they accumulated with age; they were not saturated too early; and they covered a range of organ systems. We calculated a deficit-accumulation frailty index using 39 health deficit items in 5 health domains that were assessed during the screening examination: medical history (15 items), biometric or laboratory measures (8 items), physical health (2 items), psychological health (8 items), and disability (6 items). Chronic conditions were ascertained using *ICD-10* diagnosis codes (1 inpatient or 2 outpatient diagnoses) in the past year, and other items were assessed during the screening examination. Each item was scored 0 or 1 point (eTable 2 in Supplement 1). The frailty index was calculated as the proportion of health deficits present (range, 0-1.00; higher scores indicated greater frailty). We classified participants into 4 categories using frailty index cutpoints from previous studies^19,20^: robust (<0.15), prefrail (0.15 to 0.24), mildly frail (0.25 to 0.34), and moderately to severely frail (≥0.35) categories. The following characteristics were obtained from the examination: sex, annual income level (1-20 quantiles), insurance status (employee insurance, self-employed insurance, or medical aid for low income), residential area (capital area, metropolitan area, or rural area), alcohol consumption (none, moderate level [≤1 drink per day for women and ≤2 drinks per day for men], above moderate level [>1 drink per day for women and >2 drinks per day for men], or unknown), smoking status (never, former, current, or unknown), and examination year.

### Outcome Measurements and Follow-up

The primary outcome was all-cause death. Vital status was extracted from the Korean National Health Insurance eligibility files. Secondary outcomes were (1) 8 age-related major chronic diseases (congestive heart failure, coronary artery disease, stroke, type 2 diabetes, cancer excluding nonmelanoma skin cancer, dementia, fall, and fracture) identified using relevant *ICD-10* codes (1 inpatient or 2 outpatient diagnoses) (eTable 3 in Supplement 1) and (2) disability qualifying for long-term care services from the national disability registration system. Disability type and grade were determined by the National Pension Service committee, which consisted of 2 medical specialists and a social worker, based on clinical documentations (disability certificate, medical record, and test results) and video evaluation. The results of the committee’s evaluation are updated in the Korean National Health Insurance database. Follow-up began on the day after the screening examination until the earliest of date of death, the occurrence of relevant age-related conditions, 10 years from the screening examination, or December 31, 2019.

### Statistical Analysis

Data were analyzed from October 1, 2020, to January 2022. Characteristics of the participants with different frailty levels were summarized as means (SDs), medians (IQRs), and proportions and compared using analysis of variance, Kruskal-Wallis test, or χ^2^ test. We examined 10-year cumulative incidence of all-cause death, age-related chronic conditions, and disability by frailty category. For all-cause death, we estimated hazard ratios (HR) and 95% CIs using Cox proportional hazards models. For age-related conditions and disability, we restricted analyses to those who were free of the respective condition at the time of the screening examination. To account for competing risk by death, we estimated cause-specific HRs (more relevant for etiologic research) and Fine-Gray subdistribution HRs (more relevant for estimation research) and their 95% CIs.^21^ We conducted sensitivity analysis using the frailty index as continuous variable (0 to 1.00) for all-cause death, age-related chronic conditions, and disability. All models adjusted for sex, annual income, insurance status, residential area, alcohol consumption, smoking status, and examination years. In addition, we estimated the rate of acquiring new age-related conditions by dividing the number of newly diagnosed conditions by the follow-up time at the individual level and then calculated the mean rate over those within the same frailty category. This acquisition rate has been proposed as a measure of the aging process.^22^ Analyses were performed using SAS, version 7.1 (SAS Institute Inc) and R, version 3.3.3 (R Project for Statistical Computing). Two-sided *P* < .05 was considered statistically significant.

## Results

### Characteristics of Study Population

The study population of 968 885 participants had 517 052 women (53.4%) and 451 833 (46.6%) men. The majority of participants were robust (65.2%) or prefrail (28.2%); only a small fraction were mildly frail (5.7%) or moderately to severely frail (1.0%). The mean (SD) frailty index was 0.13 (0.07) in the total population, 0.12 (0.07) in men, and 0.15 (0.07) in women (eFigure 2 in Supplement 1). Compared with participants in the robust group (n = 631 320), those in the moderately to severely frail group (n = 9215) were more likely to be women (47.8% vs 61.7%), receiving medical aid insurance for low income (2.1% vs 18.9%), and less active (median, 657 [IQR, 219-113] vs 319 [IQR, 0-693] metabolic equivalent task [min/wk]) (Table 1). They were more likely to have chronic diseases, such as congestive heart failure (1.0% vs 12.5%), diabetes (11.5% vs 53.4%), hypertension (32.1% vs 77.8%), and stroke (3.9% vs 37.1%); less desirable mean biometric or laboratory measures, such as estimated glomerular filtration rate (80.7 [25.2] to 72.5 [33.1] mL/min/1.73 m^2^), fasting blood glucose level (101.4 [24.1] to 118.0 [43.0] mg/dL [to convert to mmol/L, multiply by 0.0555]), and systolic blood pressure (126.9 [15.0] to 131.2 [16.9] mm Hg); slower mean 3-m timed up-and-go time (8.2 [2.8] to 13.0 [11.2] seconds); depressed mood (8.3% to 82.7%); higher mean scores on Prescreening Korean Dementia Screening Questionnaires (0.4 [0.6] to 2.7 [1.6] points [scores range from 0-5, with higher scores indicating poorer cognitive function); and more disability, such as needing help with bathing (0.2% to 34.7%) and meal preparation (0.5% to 50.6%).

**Table 1.** Characteristics of Participants in the National Screening Program for Transitional Ages Examination at 66 Years of Age

Characteristic	Participant group^a^				
	All	Frailty category			
		Robust	Prefrail	Mild	Moderate to severe
Sample size	968 885 (100)	631 320 (65.2)	273 150 (28.2)	55 200 (5.7)	9215 (1.0)
Frailty index, mean (SD)	0.13 (0.07)	0.09 (0.03)	0.19 (0.03)	0.28 (0.03)	0.41 (0.05)
Sex					
Women	517 052 (53.4)	302 056 (47.8)	173 224 (63.4)	36 087 (65.4)	5685 (61.7)
Men	451 833 (46.6)	329 264 (52.2)	99 926 (36.6)	19 113 (34.6)	3530 (38.3)
Annual income, median (IQR), quantile^b^	13 (6-17)	13 (6-17)	13 (6-17)	13 (5-17)	11 (2-6)
Medical aid insurance for low income	32 726 (3.4)	13 250 (2.1)	12 640 (4.6)	5097 (9.2)	1739 (18.9)
Residing in rural area	349 095 (36.0)	225 197 (35.7)	100 663 (36.9)	19 868 (36.0)	3367 (36.5)
Long-term care residents	41 421 (4.3)	19 604 (3.1)	15 007 (5.5)	5017 (9.1)	1793 (19.5)
Alcohol intake above moderate levels	126 714 (13.1)	89 927 (14.2)	30 581 (11.2)	5535 (10.0)	671 (7.3)
Current smoking	128 407 (13.3)	91 434 (14.5)	29 434 (10.8)	6432 (11.7)	1107 (12.0)
Physical activity, median (IQR), MET (min/wk)	594 (198-1036)	657 (219-1133)	438 (0-855)	370 (0-758)	319 (0-693)
Chronic disease					
Arthritis	351 626 (36.3)	172 573 (27.3)	141 062 (51.6)	32 699 (59.2)	5292 (57.4)
Asthma	79 403 (8.2)	31 541 (5.0)	36 032 (13.2)	10 000 (18.1)	1830 (19.9)
Cancer	40 060 (4.1)	19 792 (3.1)	15 794 (5.8)	3794 (6.9)	680 (7.4)
Chronic kidney disease	24 062 (2.5)	5956 (0.9)	12 589 (4.6)	4365 (7.9)	1152 (12.5)
Congestive heart failure	26 564 (2.7)	6370 (1.0)	14 061 (5.1)	4984 (9.0)	1149 (12.5)
Chronic obstructive pulmonary disease	111 966 (11.6)	46 948 (7.4)	49 223 (18.0)	13 327 (24.1)	2468 (26.8)
Coronary artery disease	84 575 (8.7)	28 653 (4.5)	41 363 (15.1)	12 125 (22.0)	2434 (26.4)
Depressed mood	197 851 (20.4)	52 658 (8.3)	99 929 (36.6)	37 641 (68.2)	7623 (82.7)
Diabetes	192 588 (19.9)	72 756 (11.5)	91 241 (33.4)	23 671 (42.9)	4920 (53.4)
Dysuria	129 904 (13.4)	45 742 (7.2)	57 832 (21.2)	21 174 (38.4)	5156 (56.0)
Fall	69 665 (7.2)	19 465 (3.1)	32 378 (11.9)	13 990 (25.3)	3832 (41.6)
Gait disorder	16 479 (1.7)	3929 (0.6)	7017 (2.6)	3615 (6.5)	1918 (20.8)
Hearing impairment	33 127 (3.4)	15 409 (2.4)	13 008 (4.8)	3748 (6.8)	962 (10.4)
Hypertension	419 034 (43.2)	202 845 (32.1)	170 370 (62.4)	38 654 (70.0)	7165 (77.8)
Stroke	73 807 (7.6)	24 721 (3.9)	34 540 (12.6)	11 127 (20.2)	3419 (37.1)
Vision impairment	1051 (0.1)	286 (0.05)	412 (0.2)	200 (0.4)	153 (1.7)
BMI, mean (SD)	24.3 (3.1)	24.0 (2.9)	25.0 (3.3)	25.2 (3.5)	25.1 (3.9)
EGFR, mean (SD), mL/min/1.73 m^2^	79.3 (26.11)	80.7 (25.2)	77.2 (27.2)	74.3 (27.8)	72.5 (33.1)
Fasting blood glucose level, mean (SD), mg/dL^c^	104.5 (27.2)	101.4 (24.1)	109.5 (30.3)	113.0 (33.6)	118.0 (43.0)
Hemoglobin level, mean (SD), g/dL^d^	13.71 (1.43)	13.85 (1.40)	13.49 (1.45)	13.34 (1.51)	13.14 (1.66)
Alanine aminotransferase level, mean (SD), IU/L^e^	24.61 (21.81)	23.61 (18.72)	26.36 (27.66)	26.94 (21.44)	26.65 (20.09)
Systolic blood pressure, mean (SD), mm Hg	128.3 (15.3)	126.9 (15.0)	130.7 (15.4)	131.4 (15.9)	131.2 (16.9)
Total cholesterol level, mean (SD), mg/dL^f^	195.44 (41.69)	195.10 (39.47)	196.19 (45.51)	196.13 (45.36)	192.43 (47.07)
3-m timed up-and-go test, mean (SD), s	8.5 (4.0)	8.2 (2.8)	8.9 (4.5)	9.8 (7.9)	13.0 (11.2)
Bone mineral density T-score ≤−2.5^g^	199 880 (38.7)	106 539 (35.3)	74 310 (42.9)	16 313 (45.2)	2718 (47.8)
KDSQ-P score, mean (SD)^h^	0.7 (0.9)	0.4 (0.6)	1.0 (1.0)	1.8 (1.3)	2.7 (1.6)
Activities requiring assistance					
Bathing	19 780 (2.0)	1563 (0.2)	6567 (2.4)	8457 (15.3)	3193 (34.7)
Dressing	20 374 (2.1)	898 (0.1)	6391 (2.3)	9078 (16.4)	4007 (43.5)
Eating	19 912 (2.1)	980 (0.2)	6397 (2.3)	8860 (16.1)	3675 (39.9)
Toileting	20 624 (2.1)	467 (0.1)	6021 (2.2)	9485 (17.2)	4651 (50.5)
Walking around house	22 712 (2.3)	1490 (0.2)	6748 (2.5)	9648 (17.5)	4826 (52.4)
Meal preparation	25 640 (2.6)	3199 (0.5)	8023 (2.9)	9756 (17.7)	4662 (50.6)

Abbreviations: BMI, body mass index (calculated as weight in kilograms divided by height in meters squared); EGFR, estimated glomerular filtration rate; KDSQ-P, Prescreening Korean Dementia Screening Questionnaire; MET, metabolic equivalent task.

^a^ Frailty index ranges from 0 to 1. Categories were defined as robust (frailty index <0.15), prefrail (0.15-0.24), mildly frail (0.25-0.34), and moderate to severely frail (≥0.35) from the screening examination at age 66 years. Unless otherwise indicated, data are expressed as No. (%) of participants. Percentages have been rounded and may not total 100.

^b^ Quantiles range from 1 (lowest) to 20 (highest).

^c^ To convert to mmol/L, multiply by 0.0555.

^d^ To convert to g/L, multiply by 10.0.

^e^ To convert to μkat/L, multiply by 0.0167.

^f^ To convert to mmol/L, multiply by 0.0259.

^g^ Bone mineral density was measured only for women.

^h^ Scores range from 0 to 5, with higher scores indicating worse cognition.

### Frailty Index at 66 Years of Age and 10-Year Risk of Death and Age-Related Conditions

**Figure 1.**
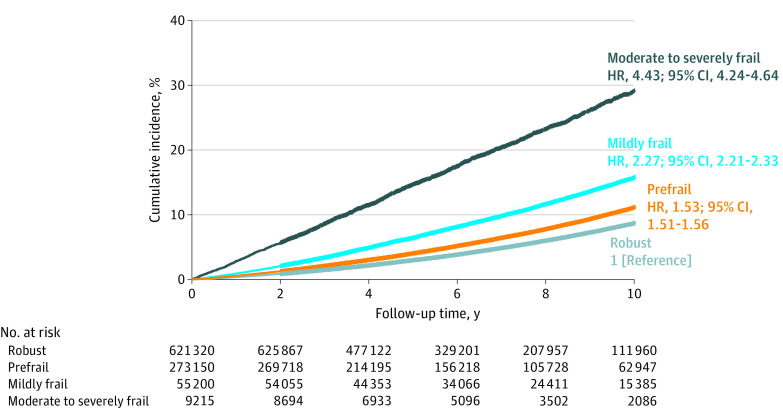
Frailty Status at 66 Years of Age and Cumulative Incidence of Death Over 10 Years Frailty categories were defined as robust (frailty index <0.15), prefrail (0.15-0.24), mildly frail (0.25-0.34), and moderately to severely frail (≥0.35) from the screening examination at 66 years of age. Hazard ratios (HRs) and 95% CIs were estimated from Cox proportional hazards models, after adjusting for sex, annual income, insurance status, residential area, alcohol consumption, smoking status, and examination years.

Over a mean (SD) follow-up of 6.7 (3.0) years, 61 783 (6.4%) participants died, including 32 683 (5.2%) in the robust group, 20 389 (7.5%) in the prefrail group, 6616 (12.0%) in the mildly frail group, and 2095 (22.7%) in the moderately to severely frail group. The corresponding incidence rates (per 100 person-years) were 0.79 in the robust group (reference group), 1.07 in the prefrail group (adjusted HR, 1.53 [95% CI, 1.51-1.56]), 1.63 in the mildly frail group (adjusted HR, 2.27 [95% CI, 2.21-2.33]), and 3.36 in the moderately to severely frail group (adjusted HR, 4.43 [95% CI, 4.24-4.64]). The 10-year cumulative incidence of death was 8.7% in the robust group, 11.1% in the prefrail group, 15.7% in the mildly frail group, and 29.2% in the moderately to severely frail group (Figure 1).

**Figure 2.**
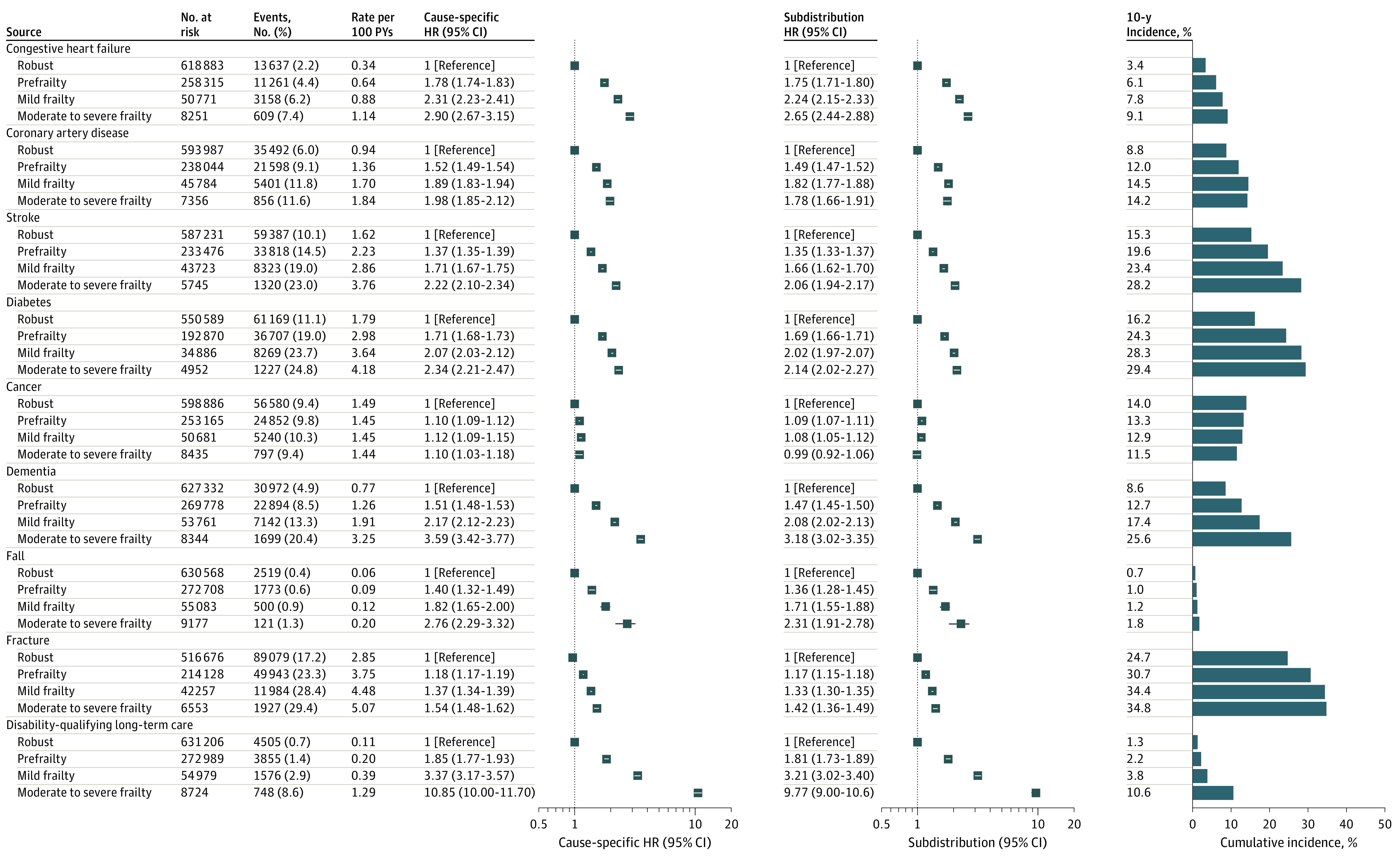
Frailty Status at Age 66 Years and Development of New Age-Related Conditions Over 10 Years Frailty categories were defined as robust (frailty index <0.15), prefrail (0.15-0.24), mildly frail (0.25-0.34), and moderately to severely frail (≥0.35) from the screening examination at 66 years of age. Cause-specific hazard ratios (HRs) and Fine-Gray subdistribution HRs with their 95% CIs were calculated to account for competing risk by death from individuals at risk (ie, free of the respective conditions at the time of the screening examination at 66 years of age), after adjusting for sex, annual income, insurance status, residential area, alcohol consumption, smoking status, and examination years. Cause-specific hazard measures the rate of disease development among those who are still alive and do not yet have the disease. Subdistribution hazard measures the risk of disease development among those who have did not have the disease and died before its onset.

Among those free of each age-related condition and comparing the robust group with the moderate to severe frailty group, frailty was associated with increased rates of developing new congestive heart failure (0.34 vs 1.14 per 100 person-years; adjusted cause-specific HR, 2.90 [95% CI, 2.67-3.15]), coronary artery disease (0.94 vs 1.84 per 100 person-years; adjusted cause-specific HR, 1.98 [95% CI, 1.85-2.12]), stroke (1.62 vs 3.76 per 100 person-years; adjusted cause-specific HR, 2.22 [95% CI, 2.10-2.34]), diabetes (1.79 vs 4.18 per 100 person-years, adjusted cause-specific HR, 2.34 [95% CI, 2.21-2.47]), cancer (1.49 vs 1.44 per 100 person-years; adjusted cause-specific HR, 1.10 [95% CI, 1.03-1.18]), dementia (0.77 vs 3.25 per 100 person-years; adjusted cause-specific HR, 3.59 [95% CI, 3.42-3.77]), fall (0.06 vs 0.20 per 100 person-years; adjusted cause-specific HR, 2.76 [95% CI, 2.29-3.32]), fracture (2.85 vs 5.07 per 100 person-years; adjusted cause-specific HR, 1.54 [95% CI, 1.48-1.62]), and disability-qualifying long-term care services (0.11 vs 1.29 per 100 person-years; adjusted cause-specific HR, 10.85 [95% CI, 10.00-11.70]) (Figure 2). Similarly, frailty was associated with greater 10-year cumulative incidence of all the outcomes, except for the cumulative incidence of cancer, which did not increase with frailty (14.0% to 11.5%; adjusted subdistribution HR, 0.99 [95% CI, 0.92-1.06]). In sensitivity analyses, an increase in frailty index was associated with an increased rate of all the outcomes (eTable 4 in Supplement 1).

### Frailty Index at 66 Years of Age and Rate of Acquiring New Age-Related Conditions

Participants acquired a mean (SD) of 0.17 (0.55) age-related conditions per year (Table 2). The mean number of newly acquired age-related conditions per year for the robust group was 0.14 (0.32); for the prefrail group, 0.23 (0.88); for the mildly frail group, 0.29 (0.44); and for the moderately to severely frail group, 0.45 (0.87). The rate was lower among women and those with higher annual income, self-employed insurance, residence in the capital area (vs metropolitan or nonmetropolitan areas), higher alcohol consumption, no history of smoking, and examination year in 2007 to 2009 (vs 2010-2013 and 2014-2017).

**Table 2.** Frailty Status at 66 Years of Age and Number of Newly Acquired Age-Related Conditions per Year During 10-Year Follow-up Period^a^

Subgroups	No. of newly acquired conditions per year, mean (SD)				
	All	Frailty category^b^			
		Robust	Prefrail	Mild	Moderate to severe
Total population	0.17 (0.55)	0.14 (0.32)	0.23 (0.88)	0.29 (0.44)	0.45 (0.87)
Sex					
Men	0.18 (0.71)	0.15 (0.31)	0.26 (1.36)	0.33 (0.55)	0.56 (1.27)
Women	0.17 (0.36)	0.13 (0.34)	0.20 (0.39)	0.26 (0.36)	0.38 (0.48)
Annual income level, quartile					
First (lowest)	0.18 (0.52)	0.14 (0.25)	0.24 (0.83)	0.31 (0.50)	0.50 (1.20)
Second	0.17 (0.45)	0.14 (0.30)	0.23 (0.69)	0.29 (0.41)	0.43 (0.75)
Third	0.18 (0.86)	0.14 (0.47)	0.23 (1.41)	0.28 (0.46)	0.43 (0.71)
Fourth (highest)	0.16 (0.27)	0.13 (0.24)	0.21 (0.28)	0.27 (0.38)	0.41 (0.51)
Insurance status					
Employee insurance	0.18 (0.78)	0.14 (0.39)	0.23 (1.30)	0.28 (0.44)	0.42 (0.68)
Self-employed insurance	0.17 (0.42)	0.13 (0.29)	0.22 (0.61)	0.28 (0.39)	0.42 (0.70)
Medical aid for low income	0.28 (0.57)	0.19 (0.31)	0.29 (0.53)	0.37 (0.65)	0.57 (1.40)
Residential area					
Capital area	0.16 (0.74)	0.13 (0.40)	0.21 (1.22)	0.27 (0.43)	0.42 (0.74)
Metropolitan area	0.17 (0.32)	0.14 (0.29)	0.23 (0.33)	0.29 (0.39)	0.46 (0.83)
Nonmetropolitan area	0.18 (0.45)	0.15 (0.25)	0.24 (0.68)	0.30 (0.47)	0.46 (1.02)
Alcohol consumption					
None	0.18 (0.45)	0.14 (0.32)	0.23 (0.62)	0.30 (0.46)	0.48 (0.82)
Moderate levels	0.16 (0.34)	0.13 (0.31)	0.22 (0.33)	0.26 (0.28)	0.44 (1.73)
Above moderate levels	0.17 (0.28)	0.14 (0.24)	0.22 (0.35)	0.27 (0.34)	0.37 (0.43)
Unknown	0.17 (1.38)	0.13 (0.51)	0.19 (2.20)	0.23 (0.49)	0.32 (0.37)
Smoking status					
Never	0.17 (0.36)	0.13 (0.33)	0.21 (0.40)	0.27 (0.39)	0.42 (0.69)
Former	0.19 (1.04)	0.14 (0.28)	0.27 (2.01)	0.33 (0.45)	0.53 (0.89)
Current	0.20 (0.42)	0.16 (0.36)	0.27 (0.42)	0.34 (0.66)	0.53 (1.57)
Unknown	0.15 (0.37)	0.13 (0.46)	0.16 (0.17)	0.23 (0.21)	0.25 (0.17)
Examination period					
2007-2009	0.16 (0.86)	0.13 (0.38)	0.19 (1.40)	0.24 (0.44)	0.34 (0.58)
2010-2013	0.16 (0.31)	0.13 (0.27)	0.20 (0.33)	0.26 (0.36)	0.42 (0.98)
2014-2017	0.19 (0.50)	0.15 (0.34)	0.27 (0.75)	0.36 (0.50)	0.57 (0.96)

^a^ The rate was defined as the number of new age-related conditions (congestive heart failure, coronary artery disease, stroke, diabetes, cancer excluding nonmelanoma skin cancer, dementia, fall, fracture, and disability qualifying for long-term care) divided by the follow-up time at the individual level; the mean rate was then calculated over those within the same frailty category.

^b^ Robust indicates frailty index of less than 0.15; prefrail, 0.15 to 0.24; mildly frail, 0.25 to 0.34; and moderately to severely frail, 0.35 or greater from the screening examination at 66 years of age.

## Discussion

In this nationwide cohort study, we found that a frailty index measured at 66 years of age was associated with death and development of a wide range of age-related diseases and disability over the next 10 years. People with a higher frailty index at 66 years of age acquired more age-related conditions over 10 years than those with a lower frailty index. These findings support that measuring a frailty index in adults aged 66 years may provide a snapshot of an older individual’s aging trajectory and offer potential opportunities for interventions to mitigate age-related health decline.

Population-based and clinical studies have shown that the prevalence of frailty among community-dwelling older adults is approximately 10%^23^ and that frailty is associated with death, disability, health care cost, and poor treatment outcomes.^5,6,7,8,9,10^ The prevalence of frailty in our study was somewhat lower than the prevalence estimates from a previous Korean cohort^24^ and other countries^23^ owing to the inclusion of adults aged 66 years. Our study expands current knowledge in several ways. First, our study removed the effect of chronologic age by restricting the study population to people who attended the screening examination at 66 years of age. We also adjusted for socioeconomic status and lifestyle characteristics. Therefore, variation in health outcomes may be attributed to different physiologic reserve and vulnerability of individuals. Second, cause-specific hazards models showed that frailty was associated with development of new cardiovascular disease, stroke, diabetes, cancer, dementia, fall, fracture, and disability. The results from subdistribution hazards models were similar, except that the cancer incidence was not increased in the moderately to severely frail group. The high risk of death in the moderately to severely frail group effectively removed people at risk for cancer, which has been reported previously.^25^ These findings support the notion that frailty is a shared risk factor for age-related diseases and disability. Third, we observed that people with a higher frailty index at 66 years of age developed chronic diseases and disability at an accelerated rate compared with those with a lower frailty index. At a given frailty level, women and people who had higher socioeconomic status and a healthier lifestyle acquired age-related conditions at a slower rate. Our results suggest that the frailty index at 66 years of age may reflect the consequence of the interactions of lifestyle, genes, and environment on structural and functional changes in cells, tissues, and organs over 66 years of life (eFigure 3 in Supplement 1)^26^ and that social support and a healthful lifestyle may slow age-related health decline.

Measuring a frailty index at 66 years of age can provide clinicians and older adults with a foundation for integrated risk assessment and prevention to promote healthy aging. Over the years, clinical prediction models have proliferated, but the clinical utility of disease-specific prediction models is questionable for older people who become vulnerable to many diseases and adverse health outcomes due to a decline in intrinsic capacity. By assessing a frailty index and adopting interventions to improve intrinsic capacity, more effective prevention against multiple age-related chronic diseases and disability may be achieved. Such preventive strategies include exercise,^12,13^ healthful diet (eg, Mediterranean diet^27^ and intermittent fasting^28^), management of chronic conditions contributing to frailty,^29^ deprescribing potentially inappropriate medications,^30^ multicomponent interventions,^14,15,31,32^ and team-based care coordination.^33,34^ Furthermore, specific deficit patterns identified from the frailty assessment can be useful to deliver targeted interventions to impaired health domains (eg, mobility, strength, or nutrition).

The utility of measuring a frailty index is premised on the availability of information to calculate the frailty index. In Korea, the National Health Insurance Corporation invites its people to a standardized examination, including a functional status questionnaire, physical examination, screening tests for depression and dementia, and laboratory tests at government-certified clinics, hospitals, and public health centers when they turn 66 years of age. Our study demonstrates the feasibility of providing information on frailty and the risk of age-related chronic disease and disability for the entire population. In the UK, the National Health Service provides general practitioners with education, training, and resources for system-wide recognition and standardized stratification of frailty and interventions based on the frailty levels.^35^ The electronic frailty index, which is calculated from administrative codes in the UK primary care electronic health record database, is used to characterize frailty at the population scale.^36^ In the US, there are insufficient national-level, coordinated efforts to systematically identify and manage older people at risk for frailty or with frailty in clinical practice.^37^ Although there is no consensus on which health deficit items should be collected, the items used in our study could be obtained during the Medicare preventive care visit, annual wellness visits, or routine primary care visits. Alternatively, frailty measures that can be completed based on self-report or without physical performance tests (eg, Clinical Frailty Scale^38^) can be useful in a busy clinical practice setting. Lack of a national infrastructure to utilize the existing data for calculation of a frailty index represents a missed opportunity in the US. There are several validated algorithms to measure frailty using administrative claims data and electronic health records,^39^ including Medicare^40,41,42^ and Veterans Affairs data,^43^ which may be useful as a screening tool for certain clinical situations and population health monitoring.

### Limitations

Our study has a few limitations that deserve mention. First, the risk of age-related outcomes in our study population may not be generalizable to other populations with different races and ethnicities. Nonetheless, we think that the association of the frailty index at 66 years of age and age-related outcomes would be consistent across different populations. Second, we only examined a deficit-accumulation frailty index. The frailty phenotype,^44^ another widely used frailty measure that is defined based on weight loss, exhaustion, weak grip strength, slow gait, and low physical activity, could not be calculated in our study due to the lack of handgrip strength measurement. While we were able to calculate a deficit-accumulation frailty index for all participants of the National Screening Program for Transitional Ages in Korea, it is unclear whether such a frailty assessment is feasible in the absence of a standardized assessment in clinical practice. Third, certain conditions (eg, falls) were underestimated due to the low sensitivity of *ICD-10* diagnosis codes, but differential outcome ascertainment by frailty status is less likely under the universal health care system. Fourth, because we included participants of the screening examination, there might be potential selection bias due to excluding nonparticipants. However, the characteristics of participants and nonparticipants were similar (eTable 1 in Supplement 1). Thus, we believe that any bias due to the exclusion of nonparticipants would be minimal.

## Conclusions

This study offers a comprehensive examination of the utility of a frailty index to estimate new age-related chronic conditions, disability, and death, as well as the rate of acquiring these conditions in a nationally representative cohort of adults who were followed from 66 years of age. By measuring a frailty index, clinicians and individuals aged 66 years can start a conversation about health changes that come with aging and discuss an individualized plan for prevention and management. Gerontologists may use a frailty index as a surrogate outcome to test midlife interventions to reverse aging. Integration of frailty in routine clinical practice and health systems may be a fundamental step to prepare for population aging.

## Notes

Supplement 1. **eFigure 1.** Flow Diagram of Study Population **eFigure 2.** Distribution of Frailty Index at 66 Years of Age **eFigure 3.** Frailty Index at 66 Years of Age as a Predictive Factor Associated With Aging Trajectory (A) and Mechanisms of Aging (B) **eTable 1.** Characteristics of the Korean National Health Insurance Corporation Enrollees by Participation Status in the Screening Examination **eTable 2.** Components of Deficit-Accumulation Frailty Index **eTable 3.** Definition of Age-Related Conditions and Disability **eTable 4.** Frailty Index at 66 Years of Age and Development of New Age-Related Conditions Over 10 Years

Supplement 2.
Data Sharing Statement
