# Supplementary material for: Assessment of Frailty Index at 66 Years of Age and Association With Age-Related Diseases, Disability, and Death Over 10 Years in Korea
Source: JAMA Netw Open. 2023 Mar 2;6(3):e2248995. doi: 10.1001/jamanetworkopen.2022.48995 (PMC9982694; doi:10.1001/jamanetworkopen.2022.48995)
Supplement: Supplement 1. — eFigure 1. Flow Diagram of Study Population eFigure 2. Distribution of Frailty Index at 66 Years of Age eFigure 3. Frailty Index at 66 Years of Age as a Predictive Factor Associated With Aging Trajectory (A) and Mechanisms of Aging (B) eTable 1. Characteristics of the Korean National Health Insurance Corporation Enrollees by Participation Status in the Screening Examination eTable 2. Components of Deficit-Accumulation Frailty Index eTable 3. Definition of Age-Related Conditions and Disability eTable 4. Frailty Index at 66 Years of Age and Development of New Age-Related Conditions Over 10 Years [file jamanetwopen-e2248995-s001.pdf]

Supplementary Online Content

Jang J, Jung H, Shin J, Kim DH. Assessment of frailty index at 66 years of age and association with age-related diseases, disability, and death over 10 years in Korea. *JAMA Netw Open*. 2022;6(3):e2248995. doi:10.1001/jamanetworkopen.2022.48995

eFigure 1. Flow Diagram of Study Population
eFigure 2. Distribution of Frailty Index at 66 Years of Age
eFigure 3. Frailty Index at 66 Years of Age as a Predictive Factor Associated With Aging Trajectory (A) and Mechanisms of Aging (B)
eTable 1. Characteristics of the Korean National Health Insurance Corporation Enrollees by Participation Status in the Screening Examination
eTable 2. Components of Deficit-Accumulation Frailty Index
eTable 3. Definition of Age-Related Conditions and Disability
eTable 4. Frailty Index at 66 Years of Age and Development of New Age-Related Conditions Over 10 Years

This supplementary material has been provided by the authors to give readers additional information about their work.

© 2023 Jang J et al. *JAMA Network Open*.

eFigure 1. Flow Diagram of Study Population

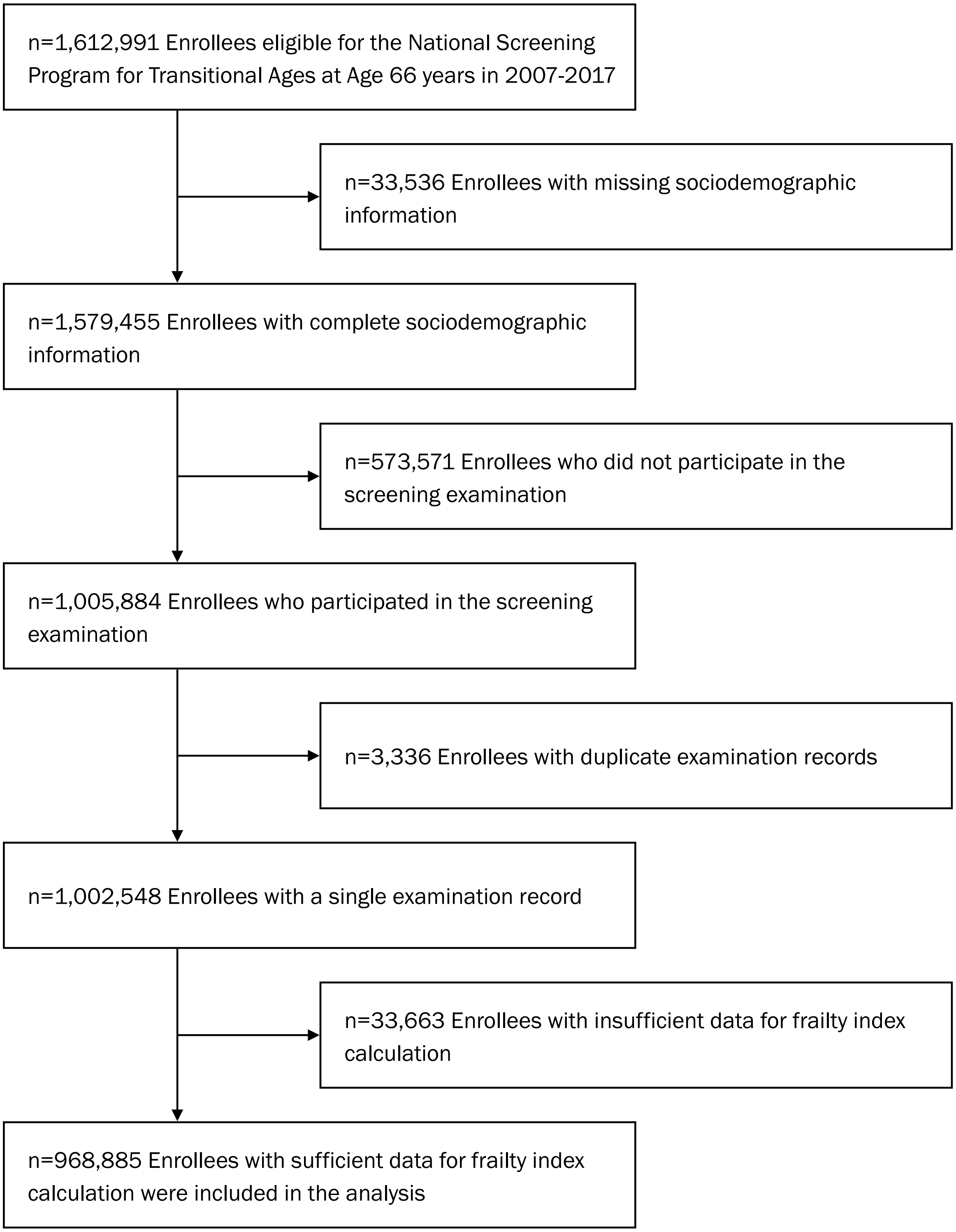

© 2023 Jang J et al. *JAMA Network Open*.

eFigure 2. Distribution of Frailty Index at 66 Years of Age

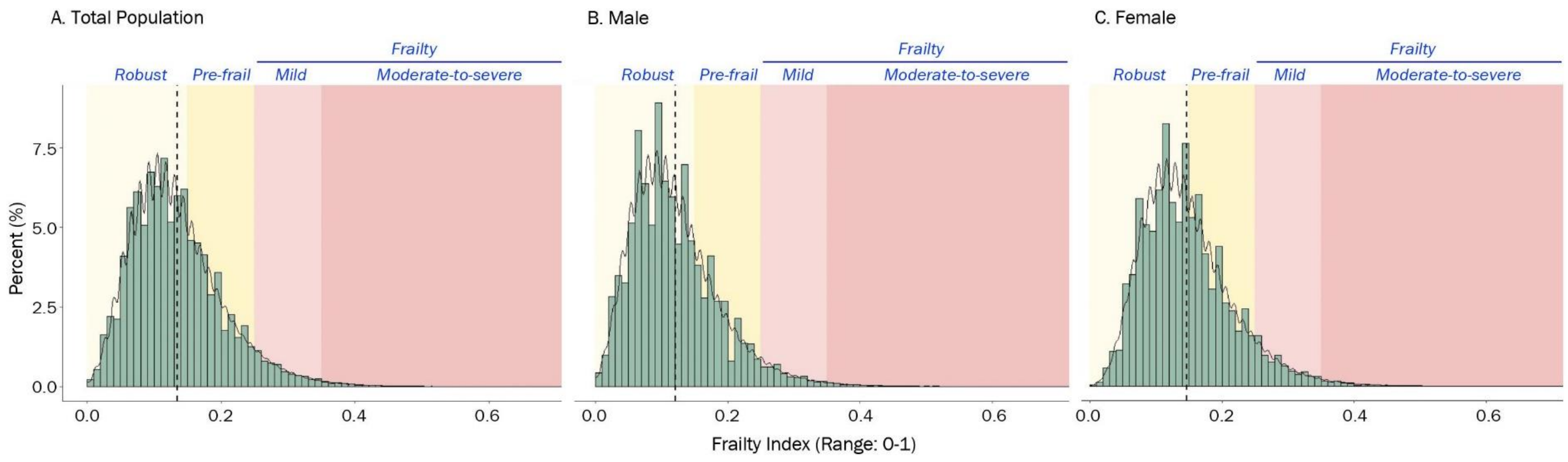

© 2023 Jang J et al. *JAMA Network Open*.

eFigure 3. Frailty Index at 66 Years of Age as a Predictive Factor Associated With Aging Trajectory (A) and Mechanisms of Aging (B)

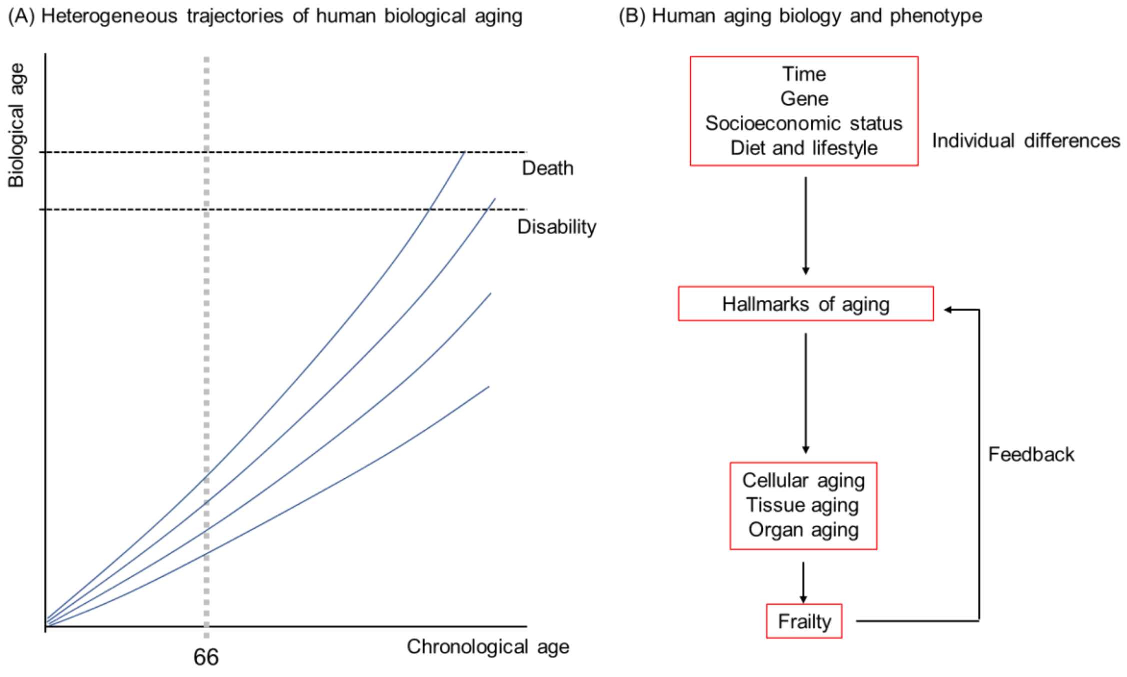

© 2023 Jang J et al. *JAMA Network Open*.

eTable 1. Characteristics of the Korean National Health Insurance Corporation Enrollees by Participation Status in the Screening Examination*

Characteristics	Non-Participants		Participants			
			Insufficient Data		Sufficient Data	
**Sample Size**	573,571	(100.0)	33,663	(100.0)	968,885	(100.0)
**Gender**						
Men	284363	(49.6)	16608	(49.3)	451833	(46.6)
Women	289208	(50.4)	17055	(50.7)	517052	(53.4)
**Annual income**						
First (lowest)	146568	(25.6)	7643	(22.7)	226594	(23.4)
Second	91356	(15.9)	5764	(17.1)	154230	(15.9)
Third	141767	(24.7)	8515	(25.3)	244764	(25.3)
Fourth (highest)	193880	(33.8)	11741	(34.9)	343297	(35.4)
**Insurance status**						
Employee insurance	199,937	(34.9)	9,828	(29.2)	289429	(29.9)
Self-employed insurance	331,974	(57.9)	23,288	(69.2)	646730	(66.7)
Medical aid for low income	41,660	(7.3)	547	(1.6)	32726	(3.4)
**Residential area**						
Capital area	245680	(42.8)	15845	(47.1)	370710	(38.3)
Metropolitan area	135538	(23.6)	6980	(20.7)	249080	(25.7)
Rural area	192353	(33.5)	10838	(32.2)	349095	(36.0)
**Examination year**						
2007	80595	(14.1)	1622	(4.8)	53911	(5.6)
2008	74825	(13.0)	845	(2.5)	86432	(8.9)
2009	51069	(8.9)	2652	(7.9)	75558	(7.8)
2010	45561	(7.9)	1211	(3.6)	77958	(8.0)
2011	39067	(6.8)	940	(2.8)	75386	(7.8)
2012	42285	(7.4)	2091	(6.2)	83430	(8.6)
2013	58002	(10.1)	2523	(7.5)	98619	(10.2)
2014	48903	(8.5)	2480	(7.4)	107047	(11.0)
2015	47206	(8.2)	6977	(20.7)	105011	(10.8)
2016	45145	(7.9)	6518	(19.4)	105316	(10.9)
2017	40913	(7.1)	5804	(17.2)	100217	(10.3)
**Disability status**						
Normal	560738	(97.8)	33,475	(99.4)	964,524	(99.5)
Mild-to-moderate disability	7632	(1.3)	146	(0.4)	3,493	(0.4)

© 2023 Jang J et al. *JAMA Network Open*.

Characteristics	Non-Participants		Participants			
			Insufficient Data		Sufficient Data	
Disability qualifying for long-term care	5201	(0.9)	42	(0.1)	868	(0.1)
**Alcohol consumption**						
None			20002	(59.4)	603562	(62.3)
Moderate levels			5570	(16.5)	160994	(16.6)
Above moderate levels			3953	(11.7)	126714	(13.1)
Unknown			4138	(12.3)	77615	(8.0)
**Smoking status**						
Never smoker			20569	(61.1)	663797	(68.5)
Former smoker			5488	(16.3)	173516	(17.9)
Current smoker			4342	(12.9)	128407	(13.3)
Unknown			3264	(9.7)	3165	(0.3)

* Data were presented in n (percent) or mean (standard deviation).

© 2023 Jang J et al. *JAMA Network Open*.

eTable 2. Components of Deficit-Accumulation Frailty Index

Components	Definition	Data Source and Measurement	Scoring
**Medical history in the past year**			
Arthritis	‘M05','M06','M08','M15','M16','M17','M19','M47','M48','M45'	Claims Data	No (0)<br>Yes (1)
Asthma	‘J45'	Claims Data	No (0)<br>Yes (1)
Cancer	‘C' except 'C44’	Claims Data	No (0)<br>Yes (1)
Chronic kidney disease	‘A1811', 'A5275', 'B520', 'C641', 'C642', 'C649', 'C689', 'D3000', 'D3001', 'D3002', 'D4100', 'D4101', 'D4102', 'D4110', 'D4111', 'D4112', 'D4120', 'D4121', 'D4122', 'D593', 'E0821', 'E0822', 'E0829', 'E0865', 'E0921', 'E0922', 'E0929', 'E102', 'E112', 'E132', 'E748', 'I120', 'I129', 'I130', 'I1310', 'I1311', 'I132', 'I701', 'I722', 'K767', 'M1030', 'M10311', 'M10312', 'M10319', 'M10321', 'M10322', 'M10329', 'M10331', 'M10332', 'M10339', 'M10341', 'M10342', 'M10349', 'M10351', 'M10352', 'M10359', 'M10361', 'M10362', 'M10369', 'M10371', 'M10372', 'M10379', 'M1038', 'M1039', 'M3214', 'M3215', 'M3504', 'N000', 'N001', 'N02', 'N03', 'N04', 'N05', 'N06', 'N07', 'N08', 'N131', 'N132', 'N1330', 'N1339', 'N14', 'N150', 'N158', 'N159', 'N16', 'N170', 'N171', 'N172', 'N178', 'N179', 'N18', 'N19', 'N250', 'N251', 'N2581', 'N2589', 'N259', 'N261', ‘'N269', 'Q6102', 'Q6111', 'Q6119', 'Q612', 'Q613', 'Q614', 'Q615', 'Q618', 'Q620', 'Q622', 'Q6210', 'Q6211', 'Q6212', 'Q6231', ' Q6232', 'Q6239', 'R944'	Claims Data	No (0)<br>Yes (1)
Congestive heart failure	‘I50','I110','I130','I132'	Claims Data	No (0)<br>Yes (1)
Chronic obstructive pulmonary disease	‘J40', 'J410', 'J411', 'J418', 'J42', 'J430', 'J431', 'J432', 'J438', 'J439', 'J440', 'J441', 'J449', 'J470', 'J471', 'J479'	Claims Data	No (0)<br>Yes (1)
Coronary artery disease	‘I20', 'I21', 'I22', 'I23', 'I24', 'I25'	Claims Data	No (0)<br>Yes (1)
Diabetes	‘E10', 'E11', 'E12', 'E13', 'E14'	Claims Data	No (0)<br>Yes (1)

© 2023 Jang J et al. *JAMA Network Open*.

Components	Definition	Data Source and Measurement	Scoring
Dysuria	Do you have dysuria?	Self-report	No (0)<br>Yes (1)
Fall	Have you fallen in the past 6 months?	Self-report	No (0)<br>Yes (1)
Gait disorder	Clinical observation of gait disorder	Clinical observation	No (0)<br>Yes (1)
Hearing impairment	Using pure-tone audiometry or whispered voice test<br>(1) Pure-tone audiometry: <40db is classified as hearing loss.<br>(2) Whispered voice test: whispers six numbers and then asks the participants to repeat. If they repeat less than three, then classified as hearing loss.	Objective measurement	No trouble (0)<br>Hearing loss on one side (0.5)<br>Hearing loss on both sides (1)
Hypertension	'H35031', 'H35032', 'H35033', 'H35039', 'I10', 'I110', 'I119', 'I12', 'I130', 'I1310', 'I1311', 'I132', 'I15', 'I674', 'N262'	Claims Data	No (0)<br>Yes (1)
Stroke	'G45', 'G46', 'I60', 'I61', 'I62', 'I63', 'I64', 'I65', 'I66', 'I67', 'I68', 'I69'	Claims Data	No (0<br>Yes (1)
Vision impairment	Visual acuity testing	Objective measurement	No trouble (0)<br>Blindness in one eye (0.5)<br>Blindness in both eyes (1)
**Biometric or laboratory measures**			
Alanine aminotransferase level, IU/L[a]		Laboratory test	≤35 (0)<br>36-45 (0.5)<br>≥46 (1)
Body mass index, kg/m^2, or weight loss		Objective measurement	18.5 to 24.9 (0)

© 2023 Jang J et al. *JAMA Network Open*.

Components	Definition	Data Source and Measurement	Scoring
			25.0 to 29.9 (0.5)<br>≥30 or ≤18.5 or weight loss 5% or more per year (1)
Bone mineral density (T-score)[b]		Diagnostic test	≥ -1.0 (0)<br>-2.4 to -1.1 (0.5)<br>≤ -2.5 (1)
Estimated GFR, ml/min/1.73m[2]		Laboratory test	≥ 60 (0)<br>30 to 59 (0.5)<br>≤30 (1)
Fasting blood glucose level, mg/dL[c]		Laboratory test	≤ 99 (0)<br>100 to 125 (0.5)<br>≥ 126 (1)
Hemoglobin level, g/dL[d]		Laboratory test	Men:<br>≥ 13.0 (0)<br>12.0 to 12.9 (0.5)<br>≤ 11.9 (1)<br>Women:<br>≥ 12.0 (0)<br>11.0 to 11.9 (0.5)<br>≤ 10.9 (1)
Systolic blood pressure, mmHg		Objective measurement	≤ 119 (0)<br>120 to 139 (0.5)<br>≥ 140 (1)
Total cholesterol, mg/dL[e]		Laboratory test	≤ 199 (0)

© 2023 Jang J et al. *JAMA Network Open*.

Components	Definition	Data Source and Measurement	Scoring
			200-239 (0.5)<br>≥ 240 (1)
**Physical health**			
Physical activity, MET-minutes/week		Self-report	≥ 1,017 (0)<br>558 to 1,016 (0.3)<br>1 to 557 (0.6)<br>0 (1)
3-m timed-up-and-go test, seconds		Objective test	≤ 10 (0)<br>11 to 19 (0.5)<br>≥ 20 (1)
**Psychological health**			
Have you dropped many of your activity or interests?		Self-report	No (0)<br>Yes (1)
Do you feel worthless the way you are now?		Self-report	No (0)<br>Yes (1)
Do you feel that your situation is hopeless?		Self-report	No (0)<br>Yes (1)
Do you think your memory is inferior to your friends or colleagues?		Self-report	No trouble (0)<br>Some (0.5)<br>A lot (1)
Do you think your memory has worsened over the last year?		Self-report	No trouble (0)<br>Some (0.5)<br>A lot (1)
Are there times when you feel memory is an impediment when doing important things?		Self-report	No trouble (0)<br>Some (0.5)<br>A lot (1)
Do others know that your memory has worsened?		Self-report	No trouble (0)

© 2023 Jang J et al. *JAMA Network Open*.

Components	Definition	Data Source and Measurement	Scoring
			Some (0.5)<br>A lot (1)
Do you think that, when performing daily activities, you have become clumsier than before?		Self-report	No trouble (0)<br>Some (0.5)<br>A lot (1)
**Disability**			
Do you take a bath or shower without assistance?		Self-report	No (1)<br>Yes (0)
Do you get dressed without assistance?		Self-report	No (1)<br>Yes (0)
If a meal is prepared, do you eat without assistance?		Self-report	No (1)<br>Yes (0)
Do you get to the toilet without assistance?		Self-report	No (1)<br>Yes (0)
Do you prepare your own meals?		Self-report	No (1)<br>Yes (0)
Do you get to places out of walking distance, such as shops, neighbors, hospitals, and government offices, without assistance?		Self-report	No (1)<br>Yes (0)

Abbreviations: GFR, glomerular filtration unite; MET, metabolic equivalent for task.

[a]To convert to µkat/L, multiply by 0.0167.

[b]Bone mineral density was only measured for women.

[c]To convert to mmol/L, multiply by 0.0555.

[d]To convert to g/L, multiply by 10.0.

[e]To convert to mmol/L, multiply by 0.0259.

© 2023 Jang J et al. *JAMA Network Open*.

eTable 3. Definition of Age-Related Conditions and Disability

Outcome	Definition*
Congestive heart failure	'I50', 'I110', 'I130', 'I132'
Coronary artery disease	'I20', 'I21', 'I22', 'I23', 'I24', 'I25'
Stroke	'G45', 'G46', 'I60', 'I61', 'I62', 'I63', 'I64', 'I65', 'I66', 'I67', 'I68', 'I69'
Diabetes	'E11', 'E12', 'E13', 'E14'
Cancer	'C' except 'C44'
Dementia	'F00', 'F01', 'F02', 'F03', 'F05', 'G138', 'G30', 'G311', 'G94', 'R4181', 'R54'
Fall	'W00', 'W01', 'W02', 'W03', 'W04', 'W05', 'W06', 'W07', 'W08', 'W09', 'W10', 'W11', 'W12', 'W13', 'W14', 'W15', 'W16', 'W17', 'W18', 'W19'
Fracture	'M80', 'S02', 'S12', 'S22', 'S32', 'S42', 'S52', 'S62', 'S72', 'S82', 'S92', 'T02', 'T08', 'T10', 'T12'
Disability qualifying for long-term care	Receiving grade 1 (very severe) or grade 2 (severe) eligibility for home and community care services

*Definition was provided using the *International Statistical Classification of Diseases and Related Health Problems, Tenth Revision* (*ICD-10*) diagnosis codes.

© 2023 Jang J et al. *JAMA Network Open*.

eTable 4. Frailty Index at 66 Years of Age and Development of New Age-Related Conditions Over 10 Years

Outcome	HR per 0.1-increase in frailty index	95% CI
Death		
Cox Proportional Hazard Models	1.58	(1.56, 1.59)
Congestive heart failure		
Cause-Specific Hazard Models	1.60	(1.58, 1.62)
Subdistribution Hazard Models	1.56	(1.54, 1.58)
Coronary artery disease		
Cause-Specific Hazard Models	1.43	(1.42, 1.45)
Subdistribution Hazard Models	1.40	(1.38, 1.41)
Stroke		
Cause-Specific Hazard Models	1.37	(1.35, 1.38)
Subdistribution Hazard Models	1.34	(1.33, 1.35)
Diabetes		
Cause-Specific Hazard Models	1.58	(1.56, 1.59)
Subdistribution Hazard Models	1.54	(1.53, 1.56)
Cancer		
Cause-Specific Hazard Models	1.08	(1.07, 1.09)
Subdistribution Hazard Models	1.06	(1.05, 1.07)
Dementia		
Cause-Specific Hazard Models	1.54	(1.52, 1.55)
Subdistribution Hazard Models	1.49	(1.47, 1.50)
Fall		
Cause-Specific Hazard Models	1.42	(1.37, 1.47)
Subdistribution Hazard Models	1.35	(1.31, 1.40)
Fracture		
Cause-Specific Hazard Models	1.19	(1.18, 1.19)
Subdistribution Hazard Models	1.16	(1.16, 1.17)
Disability qualifying for long-term care		
Cause-Specific Hazard Models	2.07	(2.02, 2.11)
Subdistribution Hazard Models	1.99	(1.95, 2.04)

Abbreviations: CI, confidence interval; HR, hazard ratio.

The association between continuous frailty index score (0 to 1) at age 66 years and outcomes was examined. Hazard ratios (95% confidence intervals) of death were estimated from Cox proportional hazards models. Cause-specific hazard ratios and Fine-Gray subdistribution hazard ratios and their 95% confidence intervals were calculated to account for competing risk by death from individuals at risk (i.e., free of the respective conditions at the time of the screening examination at age 66 years). All hazard ratios and the confidence intervals were presented for 0.1 increase in frailty index. All models adjusted for gender, annual income, insurance status, residential area, alcohol consumption, smoking status, and examination years.

© 2023 Jang J et al. *JAMA Network Open*.
