# Supplementary material for: Assessment of Frailty Index at 66 Years of Age and Association With Age-Related Diseases, Disability, and Death Over 10 Years in Korea
Source: JAMA Netw Open. 2023 Mar 2;6(3):e2248995. doi: 10.1001/jamanetworkopen.2022.48995 (PMC9982694; doi:10.1001/jamanetworkopen.2022.48995)
Supplement: Supplement 2. — Data Sharing Statement [file jamanetwopen-e2248995-s002.pdf]

Data Sharing Statement

Jang. Assessment of Frailty Index at 66 Years of Age and Association With Age-Related Diseases, Disability, and Death Over 10 Years in Korea. *JAMA Netw Open*. Published March 02, 2023. doi:10.1001/jamanetworkopen.2022.48995

Data
Data available: No
